# Functional polymorphism in miR-208 is associated with increased risk for ischemic stroke

**DOI:** 10.1186/s12920-023-01610-y

**Published:** 2023-07-31

**Authors:** Chao Liu, Yan-Ping Luo, Jie Chen, Yin-Hua Weng, Yan Lan, Hong-Bo Liu

**Affiliations:** 1 Department of Laboratory Medicine, the Second Affiliated Hospital of Guilin Medical University, Guilin, 541199 China; 2 Department of Clinical Laboratory, the Affiliated Hospital of Guilin Medical University, Guilin, 541001 China; 3 Department of Dermatology, Affiliated Hospital of Youjiang Medical College for Nationalities, Baise, 533000 China; 4 College of Medical Laboratory Science, Guilin Medical University, Guilin, 541004 China; 5 Guangxi Health Commission Key Laboratory of Glucose and Lipid Metabolism Disorders, Guilin, 541199 China

**Keywords:** Ischemic stroke, miR-208, Gene Polymorphism

## Abstract

**Background:**

The miR-208 gene is one of the microRNAs now under active studies, and has been found to play significant roles in an array of cardiovascular diseases. Nevertheless, until now, no studies have examined the relationship between the susceptibility to ischemic stroke (IS) and genetic variations in miR-208. This study explored the association between the miR-208 polymorphisms (rs178642, rs8022522, and rs12894524) and the risk of IS.

**Methods:**

A total of 205 cases of IS and 211 control subjects were included. The SNPscans genotyping test was employed to determine the genotypes of the three polymorphisms.

**Results:**

Significant correlation was observed between rs8022522 polymorphism and risk of IS on the basis of analyses of genotypes, models and alleles (GA vs. GG: adjusted OR = 2.159, 95% CI: 1.052–4.430, *P* = 0. 036; AA vs. GG: adjusted OR = 5.154, 95% CI: 1.123–23.660, *P* = 0.035; dominant model: adjusted OR = 1.746, 95% CI, 1.075–2.838, *P* = 0.025; G vs. A: adjusted OR = 2.451, 95% CI: 1.374–4.370, *P* = 0.002).

**Conclusions:**

The rs8022522 polymorphism of the miR-208 gene is significantly associated with an elevated risk of ischemic stroke in Chinese.

**Supplementary Information:**

The online version contains supplementary material available at 10.1186/s12920-023-01610-y.

## Introduction

Atherosclerosis, characterized by the progressive accumulation of lipids and plaque formation in the arteries, represents a chronic inflammatory condition involving the wall of large- and medium-sized arteries [1, 2]. The primary risk factor for culpable for the development and progression of vascular atherosclerosis is abnormal blood lipid metabolism [3]. Atherosclerotic plaque can restrict blood flow, resulting in serious conditions, such as coronary artery disease and stroke. Globally, stroke represents a leading cause of mortality and disability. Each year, 1.5 million people die of stroke and 2.5 million new cases are reported in China [4]. About 73-87% of strokes were of the most prevalent form, i.e., ischemic stroke (IS) [5]. In addition to hereditary causes, risk factors for IS included hypertension, hyperlipidemia, diabetes, smoking, drinking, and obesity [6–9]. IS has been an subject of active investigations worldwide since its staggering rates of mortality, disability, and mortality pose tremendous danger on the health and quality of life of people [10, 11].

MicroRNAs (miRNAs) are a subset of non-coding endogenous and small RNAs, consisting of approximately 20 to 26 nucleotides that regulate gene expression by repressing the translation of transcriptional products [12]. Currently, various miRNAs have been shown to be involved in the development of IS [13, 14]. For example, miR-181c was shown to inhibit the release of TNF, leading to the reduced microglial activation and neuronal cell death [15]. miR-150-3p could reportedly enhance the protective effect of neural stem cell exosomes against hypoxic-ischemic brain injury by controlling the expression of CASP2 [16]. Moreover, prior studies have shown that miR-208 expression was elevated in atherosclerosis, a pathological event underlying multiple diseases, such as myocardial infarction and IS. miR-208a was upregulated in simulated ischemic reperfusion (I/R) injured cells in vitro [17]. GO term enrichment analysis revealed that miR-208a-3p was implicated in the response to changing oxygen levels and multiple metabolic pathways, indicating that this miRNA might play a pivotal role in cellular energetics [18]. Based on these previous findings, we are led to speculate that miR-208 might be involved in the development of IS.

Prior studies also demonstrated that the susceptibility of an individual to IS may be influenced by miRNAs-related single nucleotide polymorphisms (SNPs). The risk variants included rs9301654 GA/AA genotype in miR-17-92 [19], rs4938723 TT genotype in the miR-34b/c [20] and rs2682818 GT/TT in the miR-618 [13]. The correlation between the miR-208 polymorphisms and risk of IS has not been investigated yet. In this study, we looked into whether the miR-208 polymorphisms were linked to the development of IS by utilizing a case-control approach.

## Methods

### Study subjects

The study recruited 211 healthy subjects serving as controls and 205 IS patients from the Affiliated Hospital of Guilin Medical University in Guangxi, China, between March 2020 and July 2021. The patients were diagnosed by two experienced neurologists based on clinical symptoms, physical examination and magnetic resonance imaging (MRI) or/and cranial computed tomography (CT). The diagnostic criteria were as follows: [1] focal neurological deficits [2]. focal lesions or signs/symptoms persisting for more than 24 h [3]. exclusion of non-vascular causes [4]. exclusion of cerebral hemorrhage by brain CT/MRI. Patients with transient ischemic attack (TIA), hemorrhagic stroke (stroke with bleeding), cerebral vascular malformation (CVM), cardiogenic thrombosis (CVT), malignancies, and those on lipid-lowering medications were excluded. During the same period, controls were selected from the hospital’s health care center and matched to the patients in terms of age and gender. Individuals with autoimmune diseases (e.g., SLE), blood disorders (e.g., leukemia) and cardiovascular diseases were excluded. Age, gender, total cholesterol (TC), triglycerides (TG), high density lipoprotein cholesterol (HDL-C), low density lipoprotein cholesterol (LDL-C), apolipoprotein A1 (Apo-A1), and apolipoprotein B (Apo-B) were also collected from the medical records of the patients. The subjects were all local people who had been living in Guangxi province. The study was conducted in strict accordance with the requirements of and approved by the ethics committee of the institution.

### DNA extraction and SNP genotyping

Peripheral venous blood was taken from the subjects after an 8-hour fasting for biochemical detection and DNA extraction. Genomic DNA was extracted by using the TIANamp Blood DNAKit (TIANGEN, Beijing, China), according to the manufacturer’s instructions. Genotyping was performed on an ABI3730 Genetic Analyzer (Applied Biosystems, CA, USA). 10% of the DNA samples were randomly chosen for repeat testing in order to check the experiment’s reproductivity, and the agreement rate of the results was > 99%.

### SNP selection

The selection conditions for SNPs were as follows: [1] tagSNPs in miR-208 gene [2]. SNPs in the miR-208 gene’s promoter region [3]. In silico predicted potentially-functional SNPs in the miR-208 gene [4]. greater than 5% minor allele frequency in the Chinese Han population. Finally, three SNPs were selected for further analysis, including rs178642, rs8022522, and rs12894524.

### Statistical analysis

All of the statistical analysis was conducted by using the SPSS software package, version 20.0 (SPSS, Chicago, USA). To compare continuous variables (Mean ± SD), such as clinical data, the Student’s t-test was utilized. To assess Hardy-Weinberg equilibrium (HWE) and compare categorical variables, the chi-squared test was used. The risk of IS was calculated by using odds ratios (OR), 95% confidence intervals (CI), and P values upon controlling for age, gender, TC, TG, HDL-C, and LDL-C. Statistical significance was set at a *P* < 0.05.

## Results

### Clinical features of the study population

Table 1 lists the clinical features of the patients and the controls. There was no differences in sex and age between the IS and control groups. On the other hand, HDL-C and Apo-A1 levels were lower while TG, TC and Apo-B levels were significantly higher in IS patients than in controls (*P* < 0.05).

**Table 1:** Clinical features of ISpatients and the controls

Indicators	Controls, n = 211	Cases, n = 205	*P* value
Age, years (Mean ± SD)	60.11 ± 6.96	60.34 ± 6.28	0.726
Sex (males/females)	118/93	126/79	
TG (mmol/L)	1.62 ± 0.47	2.07 ± 0.82	< 0.001
TC (mmol/L)	4.02 ± 0.99	4.76 ± 0.80	< 0.001
HDL-C (mmol/L)	1.34 ± 0.23	1.01 ± 0.12	< 0.001
LDL-C (mmol/L)	2.48 ± 0.85	2.97 ± 0.80	< 0.001
Apo-A1(g/L)	1.67 ± 0.36	1.08 ± 0.99	< 0.001
Apo-B (g/L)	0.74 ± 0.34	0.88 ± 0.18	< 0.001

TG Triglyceride, Apo-A1 Apolipoprotein A1, TC Total cholesterol, HDL-C High-density lipoprotein cholesterol, LDL-C Low-density lipoprotein cholesterol, Apo-B Apolipoprotein B

### Association between IS risk and miR-208 gene polymorphisms

Table 2 shows the genotype, allelic frequencies of rs178642, rs8022522, and rs12894524, and the risk of IS. In both controls and patients, the genotype distributions were in HWE. The rs8022522GA and AA genotypes were linked to a higher risk of IS when compared to the rs8022522GG genotype (adjusted OR = 2.159, 95% CI: 1.052–4.430, *P* = 0.036; adjusted OR = 5.154, 95% CI: 1.123–23.660, *P* = 0.035; respectively). According to allele analysis, the rs8022522A allele was substantially associated with a higher risk of IS (G vs. A: adjusted OR = 2.451, 95% CI: 1.374–4.370). Additionally, we investigated how the three polymorphisms affected IS risk under dominant and recessive models, and discovered that the rs8022522 polymorphism was correlated to a significantly higher risk of IS under both models (*P* < 0.05). Nonetheless, no significant correlation was found between IS risk and further SNPs (*P* > 0.05).

**Table 2:** miR-208 polymorphism genotype distributions in the control group and IS patients

SNP	Controls n = 211 (%)		Cases n = 205 (%)	OR (95% CI)†	*P*		
rs178642							
GG	90 (42.7)	81 (39.5)		1.00 (Ref)			
GA	93 (44.0)	95 (46.3)		1.362 (0.694–2.532)	0.393		
AA	28 (13.3)	29 (14.2)		0.859 (0.333–2.215)	0.753		
AA + AG vs. GG				0.233 (0.115–0.473)	0.000		
AA vs. GG + AG				0.499 (0.084–2.956)	0.444		
G	273 (64.7)	257 (62.7)		1.00 (Ref)			
A	149 (35.3)	153 (37.3)		0.977 (0.629–1.516)	0.977		
HWE (*P* value)	0.609	0.892					
rs8022522							
GG	160 (75.8)	127 (62.0)		1.00 (Ref)			
GA	47 (22.3)	64 (31.2)		2.159 (1.052–4.430)	0.036*		
AA	4 (1.9)	14 (6.8)		5.154 (1.123–23.660)	0.035*		
AA + AG vs. GG				1.746 (1.075–2.838)	0.024*		
AA vs. GG + AG				3.541 (1.030-11.563)	0.045*		
G	367 (87.0)	318 (77.6)		1.00 (Ref)			
A	55 (13.0)	92 (22.4)		2.451 (1.374–4.370)	0.002*		
HWE (*P* value)	0.800	0.140					
rs12894524							
GG	180 (85.3)	188 (91.7)		1.00 (Ref)			
GT	29 (13.8)	16 (7.8)		0.488 (0.147–1.361)	0.157		
TT	2 (0.9)	1 (0.5)		0.426 (0.013–13.478)	0.628		
GT + TT vs. GG				0.446 (0.153–1.298)	0.138		
TT vs. GT + GG				0.463 (0.015–14.206)	0.659		
G	389 (92.2)	392 (95.6)		1.00 (Ref)			
T	33 (7.8)	18 (4.4)		0.463 (0.172–1.247)	0.128		
HWE (*P* value)	0.498	0.314					

HWE Hardy-Weinberg equilibrium, OR odds ratio, †Adjusted by age, gender, TG, LDL-C, HDL-C, and TC;

### The analysis of blood lipid levels and miR-208 gene SNPs

Table 3 displays the correlation between lipid levels and the miR-208 SNPs. In the IS group, however, no discernible correlation was revealed between these three polymorphisms and serum levels of TC, TG, HDL-C, and LDL-C (*P* > 0.05).

**Table 3:** The correlation between lipid profile and the miR-208 SNPs

Polymorphisms	n	TC mmol/L	TG mmol/L	HDL-C mmol/L	LDL-C mmol/L	Apo-A g/L	Apo-B g/L
rs178642							
GG	81	4.67 ± 0.81	2.12 ± 0.81	1.01 ± 0.12	3.06 ± 0.85	1.07 ± 0.10	0.86 ± 0.16
GA/AA	124	4.81 ± 0.79	2.04 ± 0.83	1.01 ± 0.12	2.90 ± 0.76	1.09 ± 0.10	0.89 ± 0.18
t		-1.230	0.645	0.214	1.402	-1.569	-1.155
*P*		0.220	0.520	0.831	0.162	0.118	0.249
rs8022522							
GG	127	4.74 ± 0.82	2.04 ± 0.85	1.00 ± 0.11	2.99 ± 0.83	1.07 ± 0.10	0.88 ± 0.17
GA/AA	78	4.79 ± 0.77	2.12 ± 0.77	1.03 ± 0.14	2.92 ± 0.75	1.10 ± 0.10	0.88 ± 0.18
t		-0.400	-0.634	-1.256	0.607	-1.908	-0.290
*P*		0.690	0.527	0.211	0.545	0.058	0.772
rs12894524							
GG	188	4.73 ± 0.80	2.08 ± 0.82	1.01 ± 0.12	2.95 ± 0.79	1.08 ± 0.10	0.88 ± 0.18
GT/TT	17	5.09 ± 0.68	1.95 ± 0.81	1.01 ± 0.14	3.17 ± 0.90	1.09 ± 0.08	0.86 ± 0.10
t		-1.796	0.618	-0.071	-1.080	-0.531	0.516
*P*		0.074	0.538	0.943	0.281	0.596	0.607

TC Total cholesterol, TG Triglyceride, HDL-C High-density lipoprotein cholesterol, LDL-C Low-density lipoprotein cholesterol, Apo-A1 Apolipoprotein A1, Apo-B,Apolipoprotein B

### rs8022522 polymorphism genotype distribution in various populations

Our study further examined the genotype distribution of rs8022522 polymorphism in various populations with respect to the significance of its polymorphism in the etiology of IS (Table 4). A significant difference was found in the genotype distribution of the rs8022522 polymorphism among the populations of the HapMap-CEU, HapMap-TSI, HapMap-ASW, HapMap-YRI and HapMap-LWK (*P* < 0.05). Among the HapMap-HCB, HapMap-JPT and HapMap-CHS populations, no difference was detected (*P* > 0.05).

**Table 4:** The rs8022522 polymorphism genotype distribution in various populations

Population	n	Genotype frequency(%)			Allele frequency(%)		Ethnic group
		AA	AG	GG	A	G	
Our data	211	4 (1.9)	47 (22.3)	160 (75.8)	55 (13.0)	367 (87.0)	-
HapMap-CEU^*^	99	7 (7.1)	46 (46.5)	46 (46.5)	60 (30.3)	138 (69.7)	European
HapMap-TSI^*^	107	22 (20.6)	51 (47.7)	34 (31.8)	95 (44.4)	119 (55.6)	European
HapMap-ASW^*^	99	7 (7.1)	46 (46.5)	46 (46.5)	60 (30.3)	138 (69.7)	African
HapMap-YRI^*^	108	74 (68.5)	32 (29.6)	2 (1.9)	180 (83.3)	36 (16.7)	African
HapMap-LWK^*^	99	59 (59.6)	36 (36.4)	4 (4.0)	154 (77.8)	44 (22.2)	African
HapMap-JPT	104	2 (1.9)	17 (16.3)	85 (81.7)	21 (10.1)	187 (98.9)	Asian
HapMap-CHB	103	4 (3.9)	28 (27.2)	71 (68.9)	36 (17.5)	170 (82.5)	Asian
HapMap-CHS	105	4 (3.8)	29 (27.6)	72 (68.6)	37 (17.6)	173 (82.4)	Asian

TSI Toscans in Italy, ASW African ancestry in Southwest USA, YRI Yoruba in Ibadan, Nigeria, LWK Luhya in Webuye, Kenya, JPT Japanese in Tokyo, Japan, CHB Han Chinese in Beijing, China, CHS Southern Han Chinese, China. *: *P* < 0.05

## Discussion

The pathogenesis of IS, as a cerebrovascular disease, is multifactorial and involved. Despite continued research endeavors, up till now, its pathogenesis has not been fully elucidated. The currently held view is that traditional risk factors (e.g. hypertension, diabetes, atherosclerosis, smoking and alcohol consumption) are the external inducers of IS and genetic factors are its internal causes [21, 22]. However, it is not clear what role the genetic factors play in the development of IS, which prevents clinicians from taking advantage of genetic factors in the prevention and treatment of IS.

The earlier researches on the association between IS susceptibility and genetic variants principally focused on protein-coding genes [23–25]. The relationship between IS risk and miRNA gene-related polymorphisms has become a subject of intensive research in recent years. A variety of miRNA SNPs have been found to bear association with the genetic susceptibility to IS. For example, the promoter region of the rs4705342 polymorphism of miR-143/145 gene has been revealed to be associated to a lower risk of IS. The rs4705342CC and TC genotypes were reportedly linked to a reduced IS as compared to the rs4705342TT genotype [26]. In another study, AG and GG alleles of the rs9301654 gene were discovered to be strongly related with a lower risk of developing IS [19]. However, to our knowledge, the association of miR-208 gene rs178642, rs8022522 and rs12894524 polymorphisms with IS risk has not been investigated. In this study, we found that GA and GG genotypes of the rs8022522 gene were intimately linked to an increased risk of IS. Our findings suggested that that rs802252 polymorphism of the miR-208 gene may be related to susceptibility to IS, but validation of the findings in different races is warranted. In the analyzing of the relationship between genetic polymorphisms and susceptibility to IS, comparison of lipid profile between the cases and controls can help rule out the effect of genetic polymorphisms on statistical results. Therefore, this study further compared the lipid profile in the two groups. The results showed that serum TC, TG, LDL-C and ApoB levels were significantly higher and, on the contrary, HDL-C and ApoA1 levels were significantly lower in the IS group than in the controls, suggesting that lipid levels are associated with the risk of IS.

The miR-208 gene is a multifunctional miRNA essential for a wide array of biological processes, including the development of the heart [27] and skeletal muscles [28, 29] and the occurrence of various diseases including cancer [30, 31] and acute myocardial infarction [32] in humans. It is well-established that atherosclerosis, as a significant factor, pathophysiologically underlies a great many conditions, such as coronary disease and IS. The significance of the miR-208 gene in the etiology of atherosclerosis has recently been supported by mounting evidence. miR-208 expression has been demonstrated to be elevated in atherosclerotic samples, and miR-208 overexpression has been shown to promote VSMC migration and proliferation [33]. Moreover, the miR-208 expression was definitively upregulated in neonatal rat cardiomyocytes (NRCMs) subjected to the hypoxia/ischemia (H/I) [34]. Mohammadi et al. reported that the expression of inflammation, apoptotic factors and miR-208a were higher in patients with various cardiovascular diseases (ACS, MI, arrhythmias and HF) [35]. These findings collectively showed that the miR-208 gene might play a pivotal role in the development of IS and may may serve as a target for treatment of IS.

This study is subject to a number of limitations. Firstly, analysis of the interactions between genes and the environment was not possible in the study due to a lack of information on alcohol intake. Secondly, the power of this study might be affected by selection bias since genetic polymorphisms can differ among cohorts of a population. As a result, the conclusions reached might not be extrapolated to other ethnic groups. The genotype distribution of rs8022522, as shown in Table 4, was significantly different among the HapMap-CEU, HapMap-TSI, HapMap-ASW, HapMap-YRI and HapMap-LWK populations, but not among the HapMap-HCB, HapMap-JPT and HapMap-CHS populations. Further research is needed to establish rs8022522 as a risk factor for IS by overcoming these limitations.

## Conclusion

In conclusion, the study revealed, for the first time, that the miR-208 rs8022522 polymorphism may be associated with the susceptibility to IS, and, to some extent, provided insight into the pathogenesis of IS and potentially help clinicians to find novel biomarkers and therapeutic targets. Future studies with larger-sized samples in various ethnic groupings are needed.

## Electronic supplementary material

Below is the link to the electronic supplementary material.

Supplementary Material 1

## Data Availability

The datasets generated and/or analysed during the current study are available in the zenodo repository. (10.5281/zenodo.7971257)
